# Prognostic Significance of Blood, Serum, and Ascites Parameters in Patients with Malignant Peritoneal Mesothelioma or Peritoneal Carcinomatosis

**DOI:** 10.1155/2018/2619526

**Published:** 2018-02-15

**Authors:** Shan-shan Su, Guo-qi Zheng, Wen-jie Yin, Yu-fei Liang, Ying-ying Liu, Hui Song, Ning-ning Sun, Yu-xin Yang

**Affiliations:** Department of Gastroenterology, Cangzhou Central Hospital, Cangzhou, Hebei 061001, China

## Abstract

To determine effects of the biochemical and cytological properties of blood, serum, and ascites on survival of patients with malignant peritoneal effusion (MPeE), including malignant peritoneal mesothelioma (MPeM) and peritoneal carcinomatosis (PC), we conducted a retrospective study of patients with MPeE and healthy controls. Potential prognostic factors were identified as follows: age, sex, blood neutrophil-to-lymphocyte ratio (NLR), serum parameters, ascites parameters, serum-ascites albumin gradient, and the ascites-serum LDH ratio. Compared to those of the control group, serum albumin levels were significantly lower, and the NLR and serum LDH levels were significantly higher in the MPeE group. Overall survival (OS) was longer in patients with MPeM compared to that in patients with PC. Compared with patients in the MPeM, patients with PC had higher NLRs, ascites glucose levels, serum-ascites albumin gradients, and serum LDH levels. In contrast, their ascites albumin levels and ascites-serum LDH ratios were lower. Univariate analyses indicated that the NLR, serum LDH levels, ascites LDH levels, ascites coenocyte levels, and the ascites coenocyte-to-monocyte ratios affected the OS. Multivariate analyses identified only serum and ascites LDH levels as independent prognostic factors.

## 1. Introduction

Malignant peritoneal effusion (MPeE) is a marker that frequently indicates advanced malignant disease, and malignant ascites is a grave prognostic sign. Tumors causing carcinomatosis are commonly secondary peritoneal surface malignancies as follows: ovarian, colorectal, pancreatic, and uterine. Other causes include extra-abdominal tumors originating from lymphomas, lung and breast cancer, and a small number of primary tumors such as malignant peritoneal mesothelioma (MPeM) [1]. Malignant ascites accounts for approximately 10% of cases of ascites [2]. Survival from time of diagnosis in this patient population is poor, and there are limited therapeutic options with the goal of targeting palliation to symptoms, which include abdominal pain, nausea, vomiting, and anorexia.

Palliative procedures can improve the quality of life [3]. Therefore, reliable prognostic parameters that can be easily incorporated into clinical practice are essential. At present, few studies that focus on the predictors of survival in patients with cancer are available. These studies identify age, sex, blood neutrophil-to-lymphocyte ratio (NLR), serum albumin level, serum lactate dehydrogenase (LDH) level, ascites parameters, and the serum-ascites albumin gradient that may be associated with the prognosis of patients with multiple tumors [4–8]. However, few studies focus on the above index of patients with MPeE, particularly the relationship of ascites LDH levels with the prognosis of patients with MPeE.

Here, we present research on the prognostic factors of blood, serum, and ascites of patients with malignant ascites. The present study was performed to determine whether patient survival was affected by the type of peritoneal cavity tumor and by the parameters of the blood, serum, and peritoneal fluid as well as determine the relative contribution of each of these potential predictors to survival.

## 2. Material and Methods

The Ethics Committee of Central Hospital of Cangzhou City, Cangzhou, Hebei, China, approved this study.

### 2.1. Subjects

Patients were hospitalized because of ascites, and those eligible for inclusion in the study presented with diffuse MPeM without other primary tumors; patients with PC were identified using imaging, peritoneal histopathology, and immunohistochemistry tests administered before treatment [9, 10]. Criteria for excluding subjects were as follows: cardiac failure, kidney failure, liver cirrhosis or other causes of ascites, and surgery during follow-up treatment. We conducted a retrospective analysis of biochemical data and overall survival (OS) collected from 43 patients with MPeM and 82 with PC who were treated at our hospital from January 2012 to January 2017. Thirty-two age- and gender-matched healthy subjects served as controls.

We collected information about age, sex, primary tumor site, NLR, serum albumin levels, serum LDH levels, ascites parameters (glucose, albumin, LDH, coenocyte, monocyte, and coenocyte-to-monocyte ratio), the serum-ascites albumin gradient, and ascites-serum LDH ratios. OS was measured from the dates of diagnosis to death. Censored data were used if the patient was alive or lost to follow-up. These data were obtained from clinical charts or through telephone calls to patients' relatives.

### 2.2. Statistical Analysis

Calculations were performed using SPSS version 16.0 (Chicago, IL, USA). Chi-square test was used to compare categorical data. *t*-test was used to compare normally distributed continuous variables, and continuous variables not normally distributed are expressed as medians and ranges. The nonparametric Mann–Whitney *U* test was used to compare the significant differences between two groups. Continuous variables that were not normally distributed are expressed as median values. The relationship between prognostic factors and outcomes was modeled using univariate Kaplan–Meier survival analysis. Statistical comparisons were performed using the Kaplan–Meier method with the log-rank test. The multivariate Cox regression method was used for investigating the effects of independent variables (prognostic factors) on OS. Cox regression modeling results are presented as hazard ratios (HRs) with associated 95% confidence intervals (CIs). *P* < 0.05 indicated a statistically significant difference.

### 2.3. Limitation

There are some limitations in our study. The sample size is small in some categories. The inclusion criteria are broad and there is some degree of heterogeneity in patients with different types of primary tumor, and the retrospective study design is with inherent bias. Both univariate and multivariate statistical comparisons were used in our calculation, and it is known that univariate method might overestimate the effect size. The parameters we selected were those commonly used in clinical work, and more parameters should be explored. Therefore, further prospective study with a large sample size is necessary.

## 3. Results

### 3.1. Patients' Characteristics

Table 1 presents the patients' characteristics. Among those with MPeE, 49 (39.2%) were male and 76 (60.8%) were female (male to female ratio = 1 : 1.55).

### 3.2. OS

**Figure 1:**
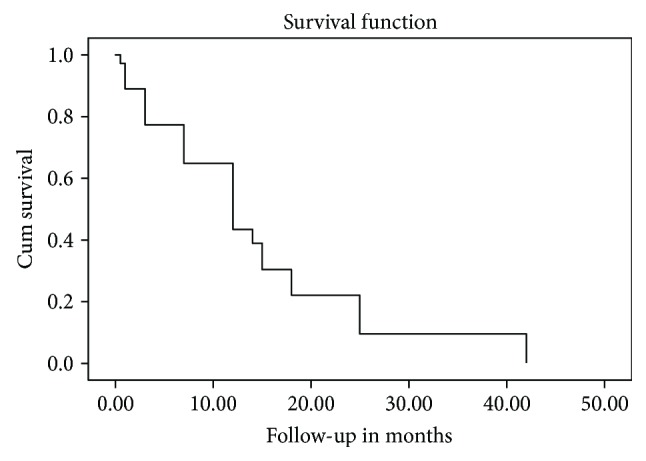
Kaplan–Meier curve showing the survival of the 125 patients. The median OS for all patients in the group was 8 months.

Table 2 and Figure 1 present the OS analysis according to the type of primary tumor. Patients had a median survival of 8 months (range, 1–42 months). Survival time was calculated in months rather than days, because months are the standard time variable used in multiple studies [11, 12]. Table 2 presents the survival analysis according to the type of primary tumor as follows: 11 months for MPeM, 9.5 months for ovarian cancer, 7 months for gastrointestinal cancer, and 6 months for liver, gall, and pancreatic cancer. Patients with MPeE associated with MPeM survived longer (11 months; range, 1–42 months) compared with those whose cancers located in other sites. Patients with liver, gall, or pancreatic cancer experienced the shortest survival (6 months; range, 1–25 months). The OS of patients with primary peritoneal carcinoma—MPeM—was significantly longer compared to the OS of those with PC (*P* = 0.034) (Table 3).

### 3.3. Serum and Ascites Albumin Levels and Serum-Ascites Albumin Gradients

Compared to those of subjects in the control group, serum albumin levels were significantly lower (*P* < 0.001) in patients with MPeE (Table 4). There was no significant difference in serum albumin between patients with MPeM and those with PC (Table 3). But serum albumin levels did not significantly affect OS according to the results of the Kaplan–Meier method and log-rank test (Table 5). The ascites albumin level was significantly higher in patients with MPeM compared to that in patients with PC (*P* = 0.046) (Table 3) and was not a significant prognostic factor (Table 5). The serum-ascites albumin gradients of patients with MPeM were lower compared to those of patients with PC (*P* = 0.002) (Table 3). In the patients with PC, there were 7 ones with massive liver metastasis, and their serum-ascites albumin gradients (17.37 ± 3.48 g/L) were especially higher compared to those of patients with MPeM (6.74 ± 3.32 g/L) (*P* < 0.001). But the serum-ascites albumin gradients of patients with MPeM were still significantly lower than those of patients with PC except massive liver metastasis ones (8.68 ± 3.60 g/L) (*P* = 0.041). However, this variable was not significantly associated with OS (Table 5).

### 3.4. Ascites Glucose Levels

Ascites glucose levels were significantly lower in patients with MPeM compared to those in patients with PC (*P* = 0.015) (Table 3). However, the ascites glucose level was not identified as a significant prognostic factor (Table 5).

### 3.5. Ascites Coenocyte and Monocyte Numbers, Coenocyte-to-Monocyte Ratios, and NLRs

**Figure 2:**
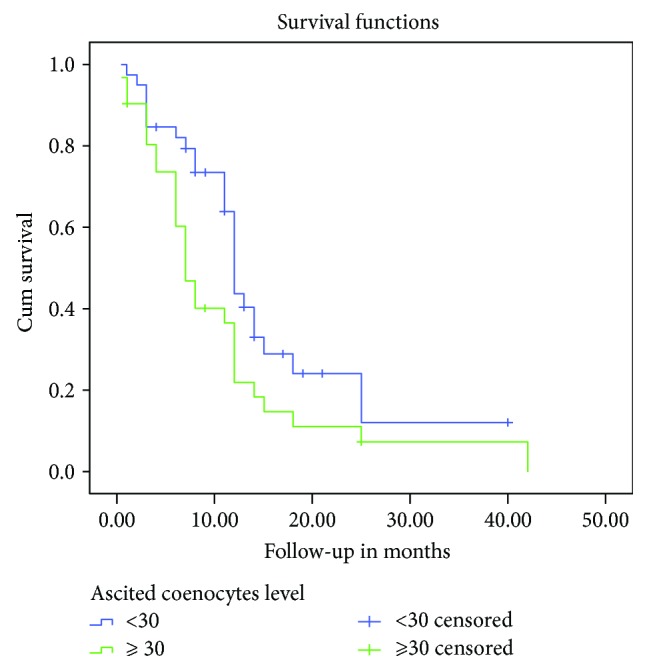
Kaplan–Meier survival curves depicting OS according to the ascites coenocyte level. The OS rate of patients with high ascites coenocyte numbers was significantly lower than that of patients with low ascites coenocyte numbers (*P* = 0.023).

**Figure 3:**
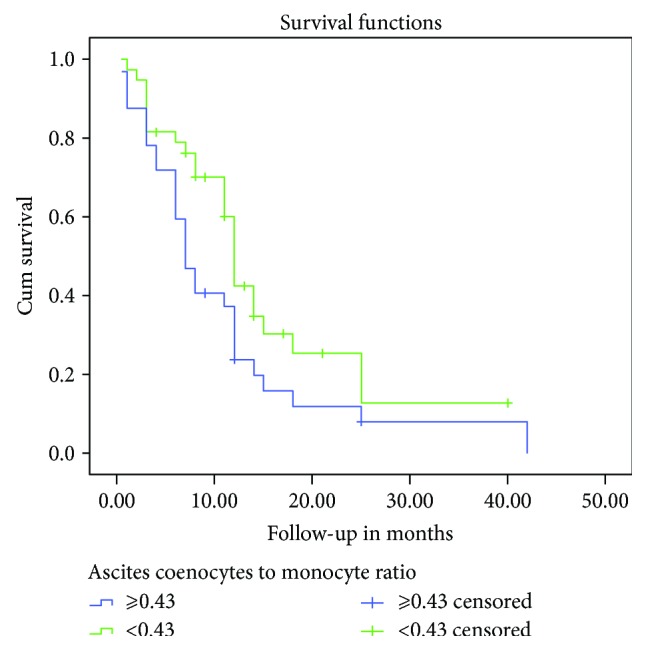
Kaplan–Meier survival curves depicting OS according to the ascites coenocyte-to-monocyte ratio. The OS rate of patients with high ascites coenocyte-to-monocyte ratio was significantly lower than that of patients with low ascites coenocyte-to-monocyte ratio level (*P* = 0.019).

The numbers of ascites coenocyte and monocyte and coenocyte-to-monocyte ratios between patients with MPeM and those with PC were not significantly different (Table 3). Kaplan–Meier univariate analysis revealed that shorter survival was significantly related to higher numbers of ascites coenocytes (*P* = 0.023) (Figure 2) and a higher ascites coenocyte-to-monocyte ratio (*P* = 0.019) (Figure 3). However, Cox proportional hazards analysis revealed that the ascites coenocyte numbers or the ascites coenocyte-to-monocyte ratio was not associated with OS (Table 5).

**Figure 4:**
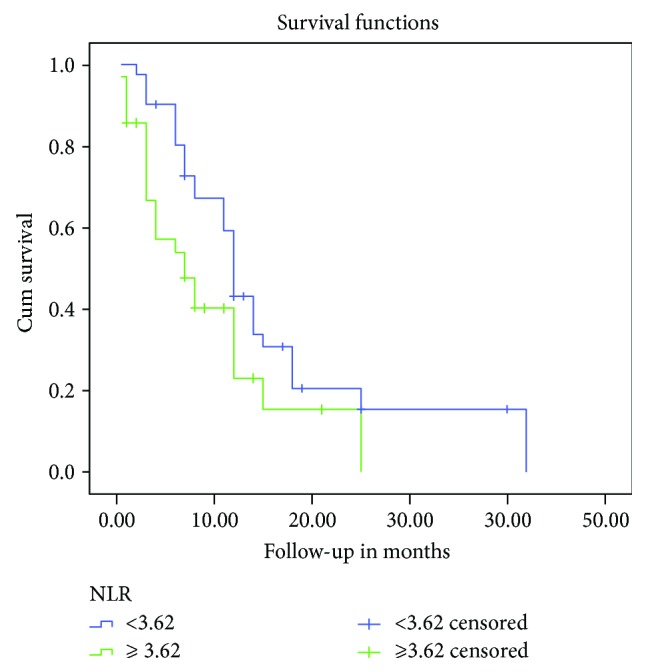
Kaplan–Meier survival curves depicting OS according to the NLR. The OS rate of patients with high NLR was significantly lower than that of patients with low NLR (*P* = 0.027).

Compared to that of healthy controls, the NLR of patients with MPeE was significantly higher (*P* < 0.001) (Table 4), and the NLR was significantly higher in patients with PC compared to that in patients with MPeM. Kaplan–Meier univariate analysis revealed that shorter survival was related to a higher NLR (*P* = 0.027) (Figure 4), although Cox proportional hazards analysis revealed that the difference was not significant (*P* = 0.770) (Table 5).

### 3.6. Serum and Ascites LDH Levels and Ascites-Serum LDH Ratios

Serum LDH levels were significantly higher in patients with MPeE (*P* = 0.020) compared to those in healthy controls (Table 4) and were significantly higher in patients with PC compared to those in patients with MPeM (*P* = 0.046) (Table 3). There was no significant difference in ascites LDH levels between patient groups (*P* = 0.239) (Table 3). The ascites-serum LDH ratios were significantly higher in patients with MPeM compared to those in patients with PC (*P* = 0.004) (Table 3).

**Figure 5:**
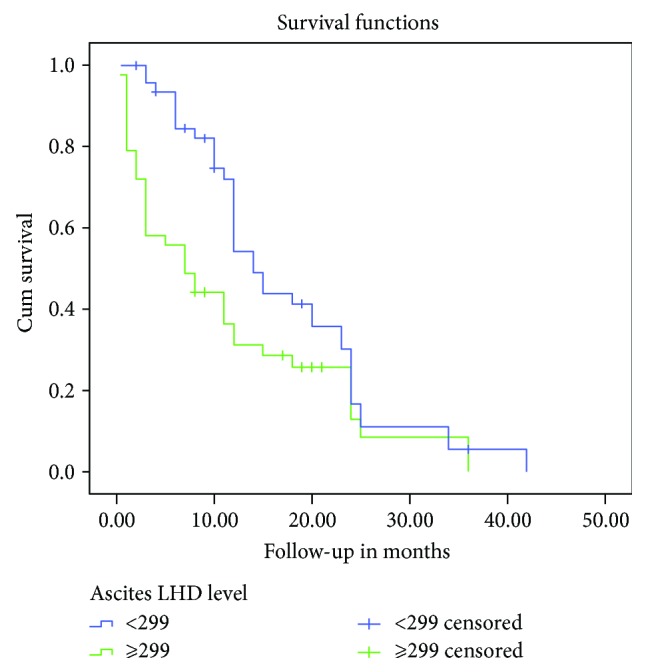
Kaplan–Meier survival curves depicting OS according to the ascites LDH level. The OS rate of patients with high ascites LDH level was significantly lower than that of patients with low ascites LDH level (*P* = 0.015).

**Figure 6:**
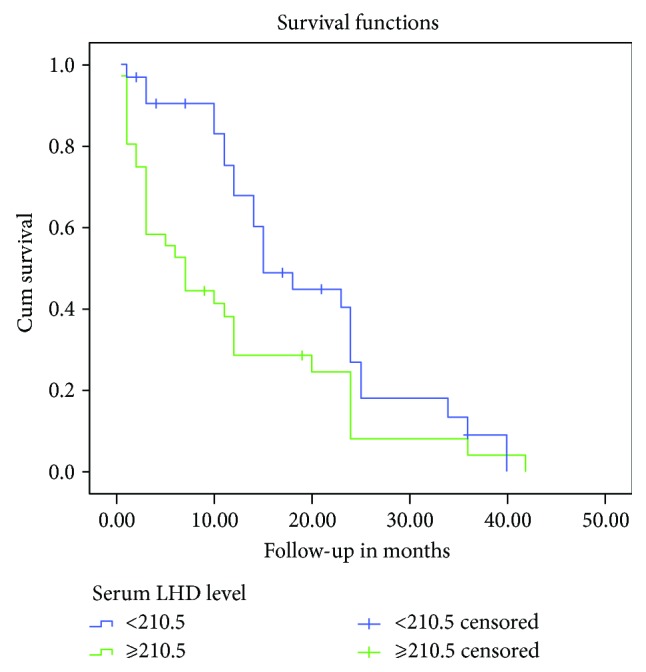
Kaplan–Meier survival curves depicting OS according to the serum LDH level. The OS rate of patients with high serum LDH level was significantly lower than that of patients with low serum LDH level (*P* = 0.008).

Kaplan–Meier univariate analysis revealed that lower OS was significantly associated with increased ascites and serum LDH levels (*P* = 0.015 and *P* = 0.008, resp.) (Figures 5 and 6) (Table 5). Ascites-serum LDH ratios were not significantly associated with OS, and Cox proportional hazards analysis revealed that ascites and serum LDH levels (*P* = 0.023 and *P* = 0.037, resp.) were independent prognostic factors associated with the OS of patients with MPeE (Table 5).

## 4. Discussion

MPeE remains one of the greatest oncologic challenges. A grim prognosis is routinely encountered with limited hope of effective treatment [13]. The TNM stage, pathological subtype, and performance status have been consistently identified as prognostic factors of cancer in clinical practice [14]. However, such factors are insufficient to guide individualized treatment of patients with MPeE. To address these problems, here we conducted a retrospective study designed to identify prognostic factors of patients with MPeE.

The diagnosis of MPeE implies poor survival with 8 months (median) in the present study. OS was significantly longer in patients with primary peritoneal carcinoma—MPeM—compared to that in patients with peritoneal metastatic carcinoma, which is consistent with the results of previous studies [15, 16].

We divided the study's subjects into two groups according to median of age, sex, blood components, serum, and ascites parameters. Female sex and age < 67 years were associated with longer survival in agreement with other studies [17], although the relationship was not statistically significant.

It is reported that the quality of patients' ascites caused by peritoneal carcinomatosis is distinct with positive cytology, high ascitic protein levels, and low serum-ascites albumin gradient [3, 18, 19]. In the present study, serum albumin levels were significantly lower in patients with MPeE compared to those in patients of the control group. Patients with MPeM had longer OS, higher ascites albumin levels, and lower serum-ascites albumin gradient compared with patients with PC. To our knowledge, PC can be associated with massive liver metastasis with a wide serum-ascites albumin gradient. We also studied the subgroup with massive liver metastasis in the patients with PC and found that the serum-ascites albumin gradients of patients with MPeM were still significantly lower than those of patients with PC except massive liver metastasis ones. However, the present study did not determine the serum and ascites albumin levels and the serum-ascites albumin gradient influenced survival time.

Cancer cells decrease the concentration of ascetic glucose [20]. Here, we found that ascites glucose levels were significantly lower in patients with MPeM compared to those in patients with PC. Low glucose concentrations in ascites were associated with an increased tumor burden, although the patients with MPeM studied here experienced longer OS. Thus, lower glucose concentrations are likely explained by the lengthy persistence a primary MPeM in the peritoneal cavity.

Many recent studies focus on inflammation in cancer. The peripheral blood NLR is a simple and valuable indicator that can reflect the magnitude of a systemic inflammatory response in patients with cancer [21, 22]. In the present study, patients with MPeE had higher NLRs compared with healthy controls, and NLRs were higher in PC patients compared to those in patients with MPeM. Kaplan–Meier univariate analysis revealed that shorter survival was associated with higher NLRs. This association can be explained by the important role of the NLR in tumor local invasion and metastasis. Here, higher percentage of neutrophils in ascites was also associated with shorter survival time, which showed that shorter OS was associated with the significant increase in the number of ascites coenocytes, accompanied by an increasing coenocytes-to-monocyte ratio.

LDH, as a regulator of hypoxia, plays a vital role in anaerobic glycolysis in cancer [23]. The serum LDH level, which is inexpensive and convenient to measure, serves as a prognostic factor of patients with malignant mesothelioma and other solid tumors [24, 25]. A high level of pleural LDH in the pleural space and its relationship with poor survival were reported in mixed cancer groups [7, 16, 26, 27]. Here, we found that compared to that in healthy controls, serum LDH was significantly higher in patients with MPeE, indicating that serum LDH is a specific diagnostic marker in patients with MPeE from benign lesions. Further, we found that patients with PC had higher serum LDH levels and experienced shorter OS compared with patients with MPeM. The reason for this phenomenon might be that PC is frequently advanced malignant disease and a grave prognostic sign accompanied by elevated serum LDH compared with MPeM.

Rare studies focus on the LDH levels in the ascites of patients with MPeE or their association with survival. Here, we found that there was no significant difference in ascites LDH levels between patients with MPeM and those with PC, although the ascites-serum LDH ratio in patients with MPeM was higher. The reason for these findings might be that MPeM is originated from peritoneum; therefore, the peritoneum tumor burden is heavy with diffused and persistent infiltration [28, 29], which is accompanied by more tissue injury induced by anaerobic glycolysis pathway and high ascites LDH levels. The major growth pattern of MPeM is peritoneal infiltration, and metastases are exceedingly rare, while the main growth pattern of PC is diffuse dissemination through the vasculature and lymph, which might account for higher serum LDH levels compared with patients with MPeM. Therefore, serum LDH is more meaningful for patients with PC, and ascites LDH is a more useful prognostic factor for MPeM.

In the present study, high levels of LDH in serum and ascites predicted poor survival, which is consistent with the use of LDH level as a significant indicator of survival outcomes [26]. Thus, LDH may be clinically applied for selecting the optimum therapeutic strategy, and patients with low levels of serum and ascites LDH may be considered suitable for measures that provide a more sustained effect. When expected survival is short, less invasive procedures should be considered (e.g., repeated abdominocentesis to relieve the symptoms). Further, inhibition of LDH appears to be promising for individualized treatment of cancers.

## 5. Conclusions

Survival time varied depending on the type of primary tumor in patients with MPeE. OS was significantly longer in patients with MPeM compared to those with PC. Serum and ascites LDH levels were independent predictors of survival of patients with MPeE. As a prognostic marker, serum LDH is more meaningful for predicting the prognosis of patients with PC and ascites LDH for predicting the prognosis of patients with MPeM. Measuring serum and ascites LDH levels may therefore be useful in clinical practice because of greater convenience and lower cost. Consideration of these factors may allow physicians to provide more precise prognoses and individualized therapeutic strategies for patients with MPeE. Future prospective randomized trials using standardized LDH cut-off values are warranted to improve statistical power.

## Figures and Tables

**Table 1:** Baseline characteristics of the study population (*n* = 125).

Characteristics	Values
Males, *n* (%)	49 (39.2)
Females, *n* (%)	76 (60.8)
Median age (range), years	67 (26–85)
Median ascites glucose level (range), mmol/L	5.2 (0.1–11.4)
Median ascites albumin level (range), g/L	28 (7–35)
Median ascites LDH level (range), U/L	299 (32–1984)
Median ascites coenocyte numbers (range), %	30 (5–99)
Median ascites monocyte numbers (range), %	70 (1–97)
Median ascites coenocyte-to-monocyte ratio (range)	0.43 (0.03–100.1)
Median serum albumin level (range), g/L	34 (15–48)
Median serum-ascites albumin gradient (range), g/L	8 (0–27.2)
Median serum LDH level (range), U/L	210.5 (115–1530)
Median ascites-serum LDH ratio (range)	1.14 (0.2–5.8)
Median blood neutrophil-to-lymphocyte ratio (range)	3.62 (0.52–33.3)

**Table 2:** Survival analysis according to primary tumor type.

Primary tumor	Patients number (%)	Median survival time months (range)
Mesothelioma	43 (34.4)	11 (1–42)
Ovary	19 (15.2)	9.5 (1–40)
Gastroenterology	26 (20.8)	7 (2–18)
Liver, gall, and pancreas	24 (19.2)	6 (1–25)
Other	13 (10.4)	6 (1–13)
Overall	125 (100.0)	8 (1–42)

**Table 3:** Survival time and potential prognostic factors in patients with mesothelioma and peritoneal carcinomatosis.

Prognostic factors	Mesothelioma (*n* = 43)	Peritoneal carcinomatosis (*n* = 82)	*P* value
Median survival time (months)	11	7.5	0.034
Sex (M/F)	16/27	33/49	0.741
Age (years)	60.7 ± 8.5	61.6 ± 12.7	0.660
Ascites glucose level (mmol/L)	4.83 ± 2.05	5.74 ± 1.96	0.015
Ascites albumin level (g/L)	28.02 ± 3.94	25.3 ± 7.21	0.046
Ascites LDH level, median (U/L)	323	252	0.293
Ascites monocyte numbers, median (%)	70	70	0.800
Ascites coenocyte numbers, median (%)	30	30	0.800
Ascites coenocyte-to-monocyte ratio	0.43	0.43	0.800
Serum albumin level (g/L)	34.14 ± 5.16	35.18 ± 5.13	0.274
Serum-ascites albumin gradient (g/L)	6.74 ± 3.32	10.06 ± 4.18	0.002
Serum LDH level, median (U/L)	191	248	0.046
Ascites-serum LDH ratio, median	2.00	1.05	0.004
Blood neutrophil-to-lymphocyte ratio, median	3.35	4.00	0.034

**Table 4:** Potential prognostic factors in healthy control and MPeE patients.

Prognostic factors	Control (*n* = 32)	MPeE patients (*n* = 125)	*P* value
Sex (M/F)	11/21	49/76	0.616
Age (years)	59.7 ± 10.2	61.1 ± 10.9	0.450
Serum albumin level (g/L)	47.51 ± 4.21	34.69 ± 5.15	<0.001
Serum LDH level, median (U/L)	179.0	210.5	0.020
Blood neutrophil-to-lymphocyte ratio, median	3.35	4.00	<0.001

**Table 5:** Univariate and multivariant analysis of the association between potential prognostic factors and survival for all 125 patients with malignant peritoneal effusions.

Prognostic factors	Categories	Patients (*n*)	Median survival time (months) (95% CI)	*P* value^a^	*P* value^b^
Sex	Male	49	8.0 (5.3–10.7)	0.131	
	Female	76	12.0 (8.9–15.1)		

Age (years)	<67	80	11.0 (8.6–13.4)	0.528	
	≥67	45	7.0 (3.5–10.4)		

Ascites glucose level (mmol/L)	<5.2	65	11.0 (8.5–13.5)	0.770	
	≥5.2	60	7.0 (1.7–12.3)		

Ascites albumin level (g/L)	<28	65	8.0 (1.4–14.6)	0.990	
	≥28	60	12.0(8.8–15.2)		

Ascites LDH level (U/L)	<299	62	14.0 (11.79–16.25)	0.015	0.023
	≥299	63	7.0 (4.4–9.6)		

Ascites coenocyte numbers (%)	<30	61	12 (11.0–13.0)	0.023	
	≥30	64	7 (5.1–8.9)		

Ascites monocyte numbers (%)	<70	55	7.0 (5.0–9.0)	0.207	
	≥70	70	12.0 (11.1–12.9)		

Ascites coenocyte-to-monocyte ratio	<0.43	64	12.0 (10.2–13.8)	0.019	
	≥0.43	61	7.0 (5.0–9.0)		

Serum albumin level (g/L)	<34	59	8.0 (4.9–11.2)	0.220	
	≥34	66	12.0 (9.6–14.4)		

Serum-ascites albumin gradient (g/L)	<8.0	62	12.0 (9.1–14.9)	0.765	
	≥8.0	63	12.0 (9.7–14.3)		

Serum LDH level (range) (U/L)	<210.5	64	14.0 (11.2–16.8)	0.008	0.037
	≥210.5	61	7.0 (4.6–9.4)		

Ascites-serum LDH ratio	<1.14	61	12.0 (6.7–17.3)	0.885	
	≥1.14	64	12.0 (10.9–13.1)		

Blood neutrophil-to-lymphocyte ratio	<3.62	62	12.0 (11.0–13.0)	0.027	
	≥3.62	63	7.0 (3.0–11.1)		

^a^Univariate analysis: Kaplan–Meier method; ^b^multivariant analysis: Cox regression method.

## Abbreviations

MPeE: Malignant peritoneal effusion
MPeM: Malignant peritoneal mesothelioma
PC: Peritoneal carcinomatosis
NLR: Neutrophil-to-lymphocyte ratio
LDH: Lactate dehydrogenase
OS: Overall survival.
